# Immediate effect of intensive atorvastatin therapy on lipid parameters in patients with acute coronary syndrome

**DOI:** 10.1186/1476-511X-9-71

**Published:** 2010-07-14

**Authors:** Dagmar Vondrakova, Petr Ostadal, Andreas Kruger

**Affiliations:** 1 Cardiovascular Center, Department of Cardiology, Na Homolce Hospital, Prague, Czech Republic

## Abstract

**Background:**

Intensive statin therapy decreases mortality and incidence of coronary events in patients after acute coronary syndrome (ACS). Recently it has been reported that spontaneous lipid levels remain clinically stable during ACS. The immediate influence of lipid levels by high-dose statin therapy initiated at admission in ACS patients is, however, not clear.

**Methods:**

We have analyzed a group of 114 patients with ACS (mean age 63.7; females 25.4%). Atorvastatin 80 mg was administered at admission and then once daily for the rest of hospitalization. The levels of total cholesterol (TC), LDL-cholesterol (LDL), HDL-cholesterol (HDL), and triglycerides (TG) were measured at admission (D0), and then every morning of hospitalization (D1, D2).

**Results:**

The mean entry values (D0) of TC, LDL, HDL and TG (in mmol/L) were 5.24, 3.26, 1.07 and 1.31, respectively. The therapy with atorvastatin 80 mg resulted in a decrease of TC levels in the first morning (D1) by 6.1% and in the second morning (D2) by 13.2% (p < 0.001 for all comparisons with the entry value D0); LDL was decreased by 5.8% (D1) and 15.6% (D2) (p < 0.001 vs. D0); the level of HDL was decreased by 7.5% (D1) and 12.1% (D2) (p < 0.001 vs. D0). In contrast, the TG level was higher in the first morning (D1) by 20.6% and in the following morning (D2) by 25.5% (p < 0.05 vs. D0).

**Conclusions:**

We have shown that intensive statin therapy started at admission in ACS patients has a highly significant, immediate effect on all monitored lipid levels. Since TC and LDL levels were decreased as predicted, reduction in HDL and increase in TG levels suggest a different acute effect of high-dose statin on lipid levels in comparison with long-term treatment of ACS patients.

## Introduction

Statins, 3-hydroxy-3-methylglutaryl coenzyme A (HMG-CoA) reductase inhibitors, have been for two decades successfully used in the therapy of hypercholesterolemia and stable ischemic heart disease. During the recent years, several large clinical trials were presented showing a beneficial effect of statin therapy started in acute coronary syndrome (ACS) patients after clinical stabilization or before discharge [1-3]. Moreover, it has been shown that intensive (high-dose) statin therapy is superior to standard-dose: in the meta-analysis intensive statin therapy decreased not only the incidence of cardiovascular events but also total mortality in patients after ACS without increase of serious side effects [4,5]. Intensive statin therapy is therefore currently widely recommended in patients after ACS.

Besides lipid-lowering effect activity, however, statins exhibit also other effects: they suppress inflammation, decrease oxidative stress, improve endothelial dysfunction, protect against ischemia damage, have a slight anti-thrombotic property etc. [6]. Statins may, therefore, favourably modulate several pathways playing an important role in the pathogenesis of ACS and these effects could be used not only in early secondary prevention after ACS but also in the therapy of unstable ACS patients at admission. Analyses of ACS registries have shown a beneficial effect of statin-therapy initiation within 24 hours of hospital admission [7] and recently also small clinical studies were published showing a promising effect of statin administered in the first-line therapy of ACS [8].

During the early phases of ACS, rapid changes occur in the serum levels of markers of myocardial necrosis, inflammation, oxidative stress, and others. Interestingly, only a limited number of authors focused on lipid levels in ACS patients; these studies which were mostly smaller in the size of population or did not use contemporary recommendations for ACS management including coronary interventions have shown a decrease in total and LDL-cholesterol [9-12]. Analysis of the baseline data from the LUNAR study has, however, demonstrated in a large ACS population treated according to current guidelines that there are only clinically insignificant spontaneous changes in the lipid parameters (total cholesterol, LDL-cholesterol, HDL-cholesterol, and triglycerides) in the first four days of ACS [13]. It is, however, still not clear whether the lipid levels in the early phase of ACS may be influenced by intensive statin treatment.

Up to the best knowledge, this is the first study focusing on the immediate effect of high-dose statin administered in the first-line therapy of ACS on the lipid parameters.

## Methods

### Subjects

The study protocol was approved by the institutional ethics committee; written informed consent was obtained from all participating subjects. One-hundred and fourteen consecutive patients admitted to the Coronary Care Unit for ACS were enrolled in this study. Eligible patients with ST elevation ACS had rest chest pain less than 12 hours before admission and ≥ 1 mm ST-segment elevation in two or more contiguous leads or new left bundle branch block on ECG. Eligible patients with non-ST elevation ACS had rest chest pain during the past 24 hours and ≥ 1 mm ST segment depression or negative T waves in two or more contiguous leads. Exclusion criteria were: concomitant active liver disease or known persistent elevation of transaminases more than three times above the upper limit, the patient is already on high-dose statin therapy, known allergy for atorvastatin or intolerance of high-dose atorvastatin, disability of oral drug administration, pregnancy or nursing, women of fertile age without effective contraception, suspicions of muscle disease, such as myositis, and subjects younger than 18 years.

At admission for ACS, blood samples were taken for examination of the baseline fasting or non-fasting serum lipid parameters: total cholesterol (TC), triglycerides (TG), LDL-cholesterol (LDL-C), and HDL-cholesterol (HDL-C); fasting lipid levels were then measured every morning of hospitalization. Atorvastatin 80 mg (Sortis, Pfizer) was administered immediately after samples for baseline lipid assessment were obtained, and then once daily for the rest of the study, if tolerated.

All patients underwent urgent coronary angiography and percutaneous coronary intervention, if necessary. Standard therapy included aspirin, heparin, low-molecular-weight heparin or fondaparinux, and clopidogrel in all patients, nitrates, beta-blockers, and angiotensin-converting-enzyme inhibitors according to the clinical conditions.

### Laboratory assays

Blood samples for lipid level analysis were taken at admission and then on the first and second morning of hospitalization. TC, LDL-C, HDL-C, and TG levels were directly measured with UNICEL DxC 800 (Beckman Coulter, USA) automatic analyzer system immediately after blood samples were obtained.

### Statistical analysis

The values are expressed as means ± standard error (SE). Statistical analysis of lipid level differences was performed by paired two-tail *t*-test. *P *< 0.05 was considered to be statistically significant.

## Results

**Figure 1:**
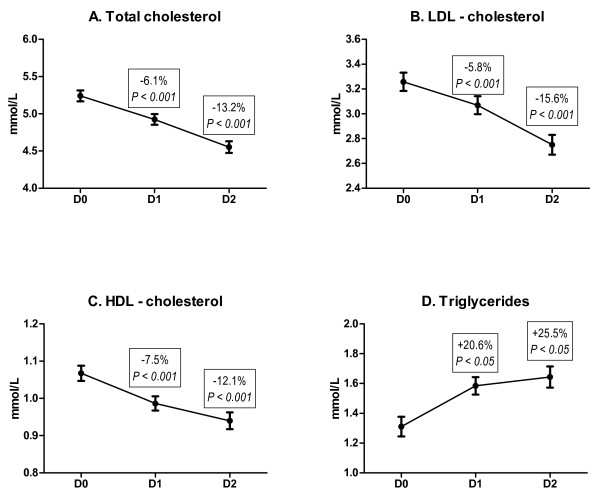
**Serum levels of lipid parameters in early phases of acute coronary syndrome in patients (N = 114) with atorvastatin 80 mg therapy initiated at admission**. Serum lipids were measured at admission (D0), first morning of hospitalization (D1), and second morning of hospitalization (D2). Panel A: levels of total cholesterol. Panel B: levels of LDL-cholesterol. Panel C: levels of HDL-cholesterol. Panel D: levels of triglycerides.

From January to December 2009 114 patients with ACS were recruited. The mean age was 63.7, majority were males (75%), ST-elevation ACS was experienced by 65% of enrolled subjects (Table 1). The mean baseline levels (D0) of TC, LDL-C, HDL-C and TG were 5.24 ± 0.07, 3.26 ± 0.07, 1.07 ± 0.02, and 1.31 ± 0.07 mmol/L, respectively. The administration of atorvastatin 80 mg resulted in a decrease of TC levels in the first morning of hospitalization (D1) to 4.92 ± 0.07 and in the second morning (D2) to 4.55 ± 0.08 (mmol/L; *P *< 0.001 for all comparisons with D0) (Figure 1A); LDL-C was decreased to 3.07 ± 0.07 (D1) and to 2.75 ± 0.08 (D2) (mmol/L; *P *< 0.001 for all comparisons with D0) (Figure 1B); the level of HDL-C was reduced to 0.99 ± 0.02 (D1) and 0.94 ± 0.02 (D2) (mmol/L; *P *< 0.001 for all comparisons with D0) (Figure 1C). In contrast, the TG level was increased to 1.58 ± 0.06 in D1 and 1.64 ± 0.07 in D2 (mmol/L; *P *< 0.05 for all comparisons with D0) (Figure 1D). The therapy with atorvastatin 80 mg was well tolerated: we did not register any case of myopathy, myositis, rhabdomyolysis or statin-related elevation of alanine aminotransferase; creatine phosphokinase and aspartate aminotransferase were elevated in most subjects as a result of myocardial necrosis.

**Table 1:** Baseline characteristics of the study group.

Mean age (years)	63.7
Female	25.0
History of	
CAD	28.0
diabetes	29.8
hypertension	67.5
Current smokers	48.2
Type of ACS	
STE	65.0
NSTE	35.0

Results are expressed in per cent if not stated differently. CAD, coronary artery disease; ACS, acute coronary syndrome; STE, ST-elevation on electrocardiogram; NSTE, non-ST-elevation on electrocardiogram.

## Discussion

The major observation of this study is that atorvastatin 80 mg significantly influences the lipid parameters already within one or two days, if administered in the first-line therapy of ACS. Furthermore, we have shown that an acute effect of intensive atorvastatin therapy on HDL-C and TG is opposite to long-term treatment: we have observed an acute decrease in HDL-C and an acute increase in TG levels.

Current evidence for the acute effect of statins on lipid levels in ACS patients is poor. Present results are in cagreement with our previous study showing rapid reduction of TC and LDL-C after administration of fluvastatin 80 mg in ACS patients [6]. A decrease of TC and LDL-C has been reported also by Michelena et al. [14] after three days of high-dose simvastatin therapy in stable high-risk patients, by Zhou et al. [15] after one-week atorvastatin therapy of ACS patients, by Marchesi et al. [16] after one-week atorvastatin therapy in women with hypercholesterolemia, and by Li et al. [17] after two weeks of simvastatin treatment in patients with dyslipidemia. On the other hand, Tsunekawa et al. [18] did not observe any difference in blood lipids after a three-day therapy with cerivastatin in elderly diabetic patients; this discrepancy can be at least partly explained by the low dose of cerivastatin (0.15 mg/day) as well as by different patient's characteristics.

In our previous study we observed only an insignificant trend to acute decrease in HDL-C as a result of fluvastatin 80 mg treatment in ACS patients and the TG level in this study was not changed [6]; this discordance with the present observation may be explained by the less potent lipid-lowering effect of fluvastatin in comparison with atorvastatin, as well as by the smaller sample size and shorter follow-up. In other studies focusing on the short-term effect of statins on the lipid profile that were carried out with stable patients and on lipid levels measured after three to fourteen days of therapy HDL-C and TG were not significantly changed [14-17]. We have no clear explanation for the marked increase in TG levels in our study, despite the fact that the baseline sample was mostly taken in non-fasting conditions whereas the D1 and D2 samples were fasting.

The major limitation of the present study is the absence of control group. It has been, however, recently reported that the spontaneous changes in lipid profile during the first days of ACS in patients managed according to current recommendations are only borderline or statistically not significant [13]; it can be, therefore, anticipated that the lipid profile in statin non-users will be similar to the baseline levels. Another limitation comes from the small size of the studied population; however, the observed changes in lipid levels are statistically highly significant. Furthermore, we have determined the lipid profile in non-fasting patients in the baseline and then in fasting conditions in D1 and D2. Non-fasting lipid measurements were, however, used also in other studies focusing on the rapid effect of statins [6,12,14] and we did not observe TG levels high enough to interfere with the analysis of other lipid parameters; moreover, surprisingly, we have found that after administration of atorvastatin the fasting TG levels were higher than the non-fasting baseline value.

It can be concluded that intensive atorvastatin therapy initiated at admission of patients with ACS has a prompt acute effect on the lipid profile and that this effect differs from the long-term statin treatment. Further research is, however, needed to confirm and explain these results.

## Competing interests

The study was supported by the grand from the Ministry of Health of the Czech Republic. Moreover, authors received research funds, consultancy honoraria, and speakers honoraria from AstraZeneca, Hoffman La Roche, Novartis, Pfizer, and Servier.

## Authors' contributions

DV: laboratory and clinical data analysis and interpretation, manuscript drafting; PO: conception and design of the trial, critical revision of the manuscript; AK: clinical data analysis and interpretation, critical revision of the manuscript.

All authors have read and approved the final manuscript.
